# Small-angle scattering from flat bilayers containing correlated scattering length density inhomogeneities

**DOI:** 10.1107/S1600576723006143

**Published:** 2023-08-16

**Authors:** Francesco Spinozzi, Leandro R. S. Barbosa, Giacomo Corucci, Paolo Mariani, Rosangela Itri

**Affiliations:** a Department of Life and Environmental Sciences, Marche Polytechnic University, Ancona, Italy; b Institute of Physics, University of São Paulo, São Paulo, Brazil; c Brazilian Synchrotron Light Laboratory (LNLS), Campinas, São Paulo, Brazil; d Institut Laue–Langevin, Grenoble, France; e École Doctorale de Physique, Université Grenoble Alpes, Saint-Martin-d’Héres, France; Australian Centre for Neutron Scattering, ANSTO, Australia

**Keywords:** small-angle X-ray scattering, SAXS, small-angle neutron scattering, SANS, domains, proteins, pores, lipid bilayers, scattering length density inhomogeneities

## Abstract

Model lipid bilayers have been widely employed as a minimal system to investigate the structural properties of biological membranes by small-angle X-ray (SAXS) and neutron scattering (SANS) techniques. These have nanometre resolution and can give information regarding membrane thickness and scattering length densities (SLDs) of polar and apolar regions. However, biological membranes are complex systems containing different lipids and protein species, in which lipid domains can be dynamically assembled and disassembled. Therefore, SLD variations can occur within the biomembrane. In this work, a novel method has been developed to simulate SAXS and SANS profiles obtained from large unilamellar vesicles containing SLD inhomogeneities that are spatially correlated over the membrane surface. Such inhomogeneities are represented by cylindrical entities with equivalent SLDs. Stacking of bilayers is also included in the model, with no correlation between horizontal and vertical order. The model is applied to a lipid bilayer containing SLD inhomogeneities representing pores, lipid domains, and transmembrane, partially immersed and anchored proteins. It is demonstrated that all the structural information from the host lipid bilayer and from the SLD inhomogeneity can be consistently retrieved by a combined analysis of experimental SAXS and SANS data through the methodology proposed here.

## Abstract 2

An approach to simulating small-angle X-ray and neutron scattering curves of planar bilayers with horizontally correlated inhomogeneities representing lipid domains, proteins or pores is presented.

## 1. Introduction

Small-angle X-ray scattering (SAXS) and small-angle neutron scattering (SANS) are known to be suitable experimental techniques to investigate, at nanometric resolution, the structure of self-assembling systems formed by amphiphilic molecules, such as lipids (the main component of biological membranes) and surfactants (the molecules at the basis of detergents and cosmetics) (Glatter & Kratky, 1982 ▸). Small-angle scattering (SAS) can provide information about the shape of the aggregated structures, which basically spans from spheres to cylinders or lamellae, their dimensions and the spatial correlation between these nanosized objects. The latter comprises the signature of their lyotropic polymorphism: for instance, the typical phases observed for lipids, which are dependent on the main chemical-physical parameters, such as concentration, temperature, pressure, pH and ionic strength, are the micellar phase (direct or inverse), the hexagonal phase (direct or inverse), the lamellar phase (including the multilamellar phase formed by vesicles) and the various types of cubic phases (Mariani *et al.*, 1988 ▸).

Concerning the study of nano-scaled model lipid bilayers (*e.g.* lipid vesicles dispersed in an aqueous environment), the advantage of SAXS is its dependence not only on the overall dimension of the vesicles but also on their internal structure. This is due to the spontaneous organization of amphiphilic molecules in two domains, the first hydrophobic, mainly formed by methylene groups CH_2_ of low electron density (which, multiplied by the classical radius of the electron *r*_e_ = 0.28 × 10^−12^ cm, gives the scattering length density, SLD) with respect to the aqueous solution, and the second polar, where the presence of electronegative atoms such as oxygen, nitrogen and phosphorus determines a high electron density. The polar domain includes hydration water molecules and, *e.g.* in the case of charged phospholipids, a fraction of counterions, both contributing to the polar domain average electron density.

The SAXS signal originates from the Fourier transform of the electron-density profile of the lipid bilayer, so that any electron-density variation is mapped onto the experimental SAXS intensity recorded as a function of the scattering angle. The analysis of SAXS data can be carried out through different levels of approximation. In the simplest approach, which is the most common reported in the literature (Luzzati, 1968 ▸; Guinier, 1963 ▸; Feigin & Svergun, 1987 ▸; Lindner & Zemb, 2002 ▸), the lipid bilayer thickness and the electron densities of both polar and hydrophobic domains are considered free parameters that can be determined by the best fit to the experimental SAXS curve. However, for many techniques based on scattering, different sets of parameters can lead to a similar SAXS signal. Therefore, some constraints must be imposed on the fitting parameters, for example exploiting the known physicochemical and structural properties of the lipids used. The combined analysis of SANS data over the same investigated self-assembled structures allows us to retrieve concomitantly the most appropriate fitting parameters from the SAS data. SANS is sensitive to the neutron scattering length density, which can be modified by controlling the degree of deuteration of water and lipids (Lindner & Zemb, 2002 ▸; Petrache *et al.*, 1997 ▸; Klauda *et al.*, 2006 ▸; Kučerka *et al.*, 2008 ▸; Pan *et al.*, 2015 ▸; De Rosa *et al.*, 2018 ▸).

When the bilayers are organized in a multilamellar phase, it is possible to extract information about the structural parameters of the lamellar stacking: the SAS diffraction peaks furnish the repetition distance (*e.g.* the total thickness of the bilayer and water layers) from the peak position and the degree of correlation between the lamella from its width.

Although the scattering intensity from bilayers made up of only one type of lipid has been widely described, it is well established that biological membranes are more complex systems. They host proteins in different ways, whether anchored in the surface, partially immersed in the hydrophobic domain or as transmembrane proteins straddling the whole thickness of the membrane. Lipid domains formed by distinct lipids can be assembled and disassembled, providing clues to the binding of specific proteins (Sezgin *et al.*, 2017 ▸). Further, antimicrobial peptides or toxins can promote pores in the membranes (Mesa-Galloso *et al.*, 2021 ▸). Because of their effects on the SLD profile, all these situations may produce different but characteristic SAS curves (Heberle *et al.*, 2013 ▸; Marquardt *et al.*, 2015 ▸; Doktorova *et al.*, 2019 ▸; Semeraro *et al.*, 2021 ▸).

Different analytical or semi-analytical models have been developed to describe the form factor of spherical vesicles containing lipid domains from scattering data. Pencer *et al.* (2005 ▸) used coarse-grained models to calculate the form factor of radially polydisperse spherical vesicles containing a single domain or different small domains. The model was applied to fit SANS data of lipid mixtures that show phase separation at low temperatures. The contrast conditions were optimized using both deuterated and hydrogenated lipids dissolved in H_2_O/D_2_O mixtures in such a way that at high temperature, when there is no phase separation, the contrast between the lipid mixture and the solvent was zero. Interestingly, applying the coarse-grained model to SANS data of unilamellar vesicles (LUV) of DOPC:DPPC:cholesterol^1^ (molar ratio 1:1:1), the authors showed that, at room temperature, each LUV displayed approximately 30 lipid domains of average radius 100 Å. A coarse-grained approach for calculating the form factor of polydisperse spherical vesicles forming lipid domains was also used by Heberle *et al.* (2013 ▸) and applied to analyse SANS data of lipid mixtures of DOPC:POPC:DSPC:chol­esterol under optimal contrast conditions, with the aim of calculating bilayer thickness and domain size. The authors obtained values of domain radius and number of domains that could vary from 68 Å and 23 domains to 225 Å for one to four domains, respectively, depending on the molar ratio of the lipids. Anghel *et al.* (2007 ▸) utilized, for the first time, the powerful spherical harmonic approach (Stuhrmann, 1970 ▸; Svergun & Stuhrmann, 1991 ▸; Spinozzi *et al.*, 1998 ▸) to calculate the form factor of a spherical vesicle containing one circular nanodomain. This approach was then extended (Heberle *et al.*, 2015 ▸) and the analytical form factor for the case of several domains of arbitrary size and spatial configuration was derived. In subsequent work, Anghel *et al.* (2018 ▸, 2019 ▸) calculated the correlation between domains in the case of vesicles containing two or three domains. For vesicles with more than three domains, they simulated the correlation between domains using a Monte Carlo method, the results of which were subsequently interpreted using a Percus–Yevick equation in spherical geometry combined with the Ornstein–Zernike relation. Dorrell *et al.* (2020 ▸) developed an advanced method to calculate the SANS curves of highly curved and fluctuating vesicles. This method combines a molecular and a continuum approach to discriminate between inner and outer leaflets of the vesicle. Recently, Krzyzanowski *et al.* (2023 ▸) have analysed SANS curves of a mixture of two lipids, DLPC and DPPC, that exhibits solidus–liquidus phase coexistence by using a bead model to calculate the form factor of polydisperse vesicles.

Most of the articles cited above concern the study, especially by SANS, of small polydisperse spherical vesicles (diameter 300–600 Å) in which there are circular domains having a constant SLD. The effects of the curvature and the total vesicle area are important constraints to establish the number and size of the domains. In this work we use a different approach. We work exclusively in planar geometry, thus assuming that the radius of the vesicle is so large that it does not show curvature effects or limit the number of domains. We describe how the SAS curves from such flat lipid bilayers are affected by the presence of SLD inhomogeneities such as those arising from pores, lipid domains and membrane proteins, and how structural information about these in­homo­geneities can be retrieved by model fitting. In particular, we simulate SAXS and SANS profiles for lipid bilayers containing DOPC and DPPC phospholipids and a certain number of laterally correlated SLD inhomogeneities (hereafter referred to as ‘islands’), defined as cylindrical entities representing lipid domains, pores, aqueous channel-forming proteins, anchored proteins, or partially immersed or transmembrane proteins, taking into account their SLDs. The resulting SLD inhomogeneity model has been integrated into the *GENFIT* software (Spinozzi *et al.*, 2014 ▸), freely available at https://sites.google.com/site/genfitweb/, and was used to fit the simulated curves. The good agreement between fitted and simulated profiles obtained in the different investigated cases confirms the robustness of the method. The model also includes the possibility of bilayer stacking with no correlation between horizontal and vertical order.

## 2. Model development

**Figure 1:**
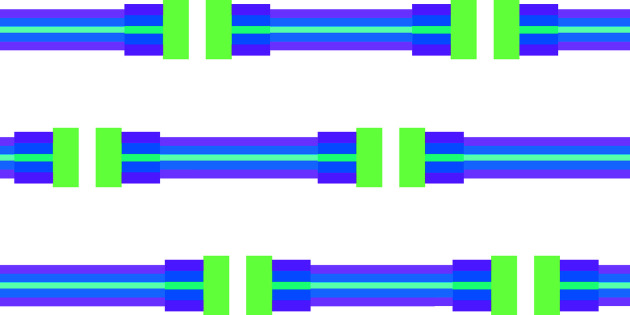
Schematic diagram of stacking of bilayers with SLD inhomogeneities (referred to as islands throughout the paper). The island-free bilayer is described by *N*_b_ = 5 levels of SLD, represented by magenta (lipid polar head group), blue (alkyl chains of the lipids), cyan (the inner part of the bilayer rich in CH_3_ groups), blue and magenta layers, in order. The island contains *N*_s_ = 3 cylindrical shells with the following levels of SLD: *N*_1_ = 0 (white hole), *N*_2_ = 1 (green layer), *N*_3_ = 5 (dark magenta, dark blue, dark cyan, dark blue and dark magenta layers).

To take into account possible SLD inhomogeneities in the SAS profiles of lipid bilayers, the following methodology has been developed. First, we consider a solution of randomly oriented stacks of *N* parallel bilayers. Associated with each bilayer are *M* identical islands, described by *N*_s_ cylindrical shells of SLD inhomogeneities with their common axis perpendicular to the bilayer surface (Fig. 1 ▸). We assume that the bilayer surface is the area of a circle with radius *R*_b_ much greater than the typical bilayer thickness.

The SLD of the system at the point 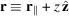 ( 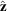 is the unit vector in the direction perpendicular to the bilayer *xy* plane) can be written as 

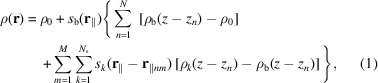

where *z*_*n*_ is the vertical displacement of the *n*th bilayer and **r**_∥*nm*_ is the two-dimensional vector in the *xy* plane of the *n*th bilayer that gives the position of the centre of the *m*th island. ρ_0_ is the solvent SLD. Referring to a bilayer placed at the centre of the reference system, the function *s*_b_(**r**_∥_) is equal to 1 when |**r**_∥_| ≤ *R*_b_ and 0 otherwise. The bilayer SLD profile along the *z* axis is described by the function ρ_b_(*z*). On the other hand, when the origin is at the centre of an island, the function *s*_*k*_(**r**_∥_) is 1 when the point **r**_∥_ belongs to the *k*th cylindrical shell of the island, whose SLD profile along the *z* axis is ρ_*k*_(*z*); otherwise it is 0. The scattering amplitude is the Fourier transform of the SLD [equation (1)] in excess with respect to ρ_0_,

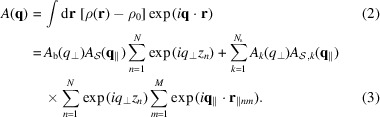

The scattering vector, defined as **q** = 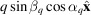 + 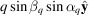 + 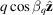 ( 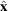 and 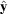 being unit vectors along the axes *x* and *y*, respectively), is also written as **q** 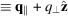 . We have introduced the following four partial amplitudes, 

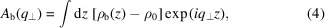




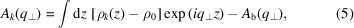




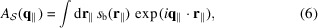




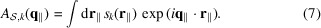


*A*_b_(*q*_⊥_) is the Fourier transform of the SLD of the bilayer without islands in excess with respect to the solvent, *A*_*k*_(*q*_⊥_) is the Fourier transform of the SLD of the *k*th cylindrical shell that exceeds the corresponding SLD of the bilayer without islands, 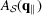 is the *xy* Fourier transform of the surface of the whole bilayer without islands, and finally 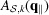 is the *xy* Fourier transform of the *k*th circular shell on the bilayer surface.

**Figure 2:**
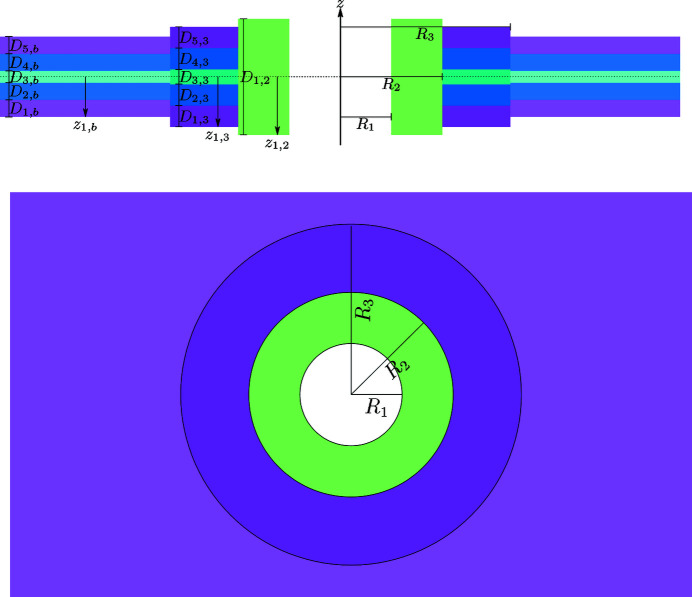
A representation of an island in a bilayer with the geometric parameters indicated. The bilayer contains *N*_b_ = 5 levels of SLD, colour coded as in Fig. 1. The island contains *N*_s_ = 3 cylindrical shells with the following levels of SLD: *N*_1_ = 0, *N*_2_ = 1, *N*_3_ = 5 (see Fig. 1 ▸ caption).

All the SLD profiles along the *z* axis, ρ_*l*_(*z*) [with *l* standing for the bilayer without islands (*l* = b) or the *k*th cylindrical shell of the island (*l* = *k*)], are modelled by an *N*_*l*_-level function, with transitions between two successive levels described by the smooth error function erf(*z*) (Spinozzi *et al.*, 2010 ▸). 

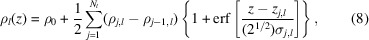

where *z*_*j*,*l*_ is the *z* coordinate of the *j*th level of the *l*th profile with thickness *D*_*j*,*l*_, 

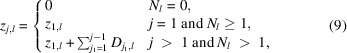

and σ_*j*,*l*_ is the smoothness of the transition between the (*j* − 1)th and the *j*th levels (Fig. 2 ▸). In equation (8), we set 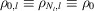 . Note that the expression above does not assume any symmetry of ρ_*l*_(*z*).

According to these assumptions, the four amplitudes in equations (4)–(7 [Disp-formula fd6] [Disp-formula fd7]) become the following analytical expressions: 

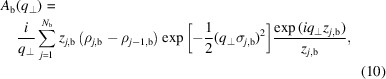




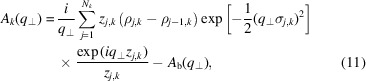




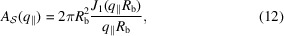




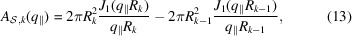

where *J*_1_(*x*) are the first Bessel functions of integer order, and *R*_*k*_ represents the radius of the *k*th cylindrical shell of the island, with *R*_0_ ≡ 0 by definition.

At **q** = 0, the asymptotic values of these functions are 

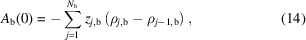




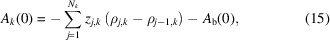




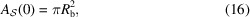




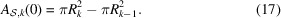

The modulus square of the scattering amplitude becomes 

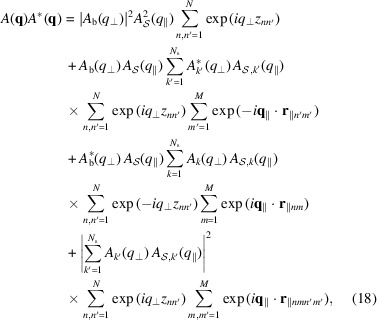

where 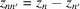 and 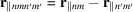 . By averaging over all possible stacks of the bilayer (*z*_*n*_) and all possible positions of islands (**r**_∥*nm*_) and by assuming that there is no correlation between vertical and horizontal order, the previous equation transforms into 

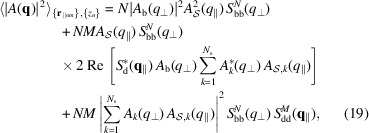

where we have introduced the 1D bilayer–bilayer structure factor, 

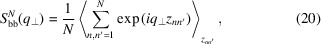

and the 2D island and island–island structure factors, 

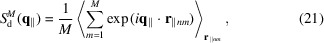




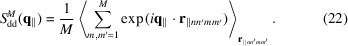

The angle brackets represent the averages over the distribution of stacking distances 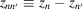 [equation (20)], island positions **r**_∥*nm*_ [equation (21)] and island–island distances 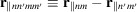 [equation (22)]. We assume that the island distribution along the *xy* bilayer plane is described by the well known paracrystal theory (PT) in two dimensions (Hosemann & Bagchi, 1952 ▸, 1962 ▸; Hosemann *et al.*, 1967 ▸; Wilke, 1983 ▸; Matsuoka *et al.*, 1987 ▸; Lazzari, 2002 ▸; Frühwirth *et al.*, 2004 ▸). Firstly, we consider that the island distribution along the *xy* bilayer plane is based on a distorted two-dimensional hexagonal lattice, with unit-cell vectors 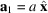 and 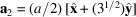 . The lattice parameter *a* represents the *average island–island distance*. Secondly, along both directions **a**_1_ and **a**_2_, we assume a unique average number, *N*_*a*_ = *M* ^1/2^, of islands with a unique distortion factor, *g*_*a*_ = σ_*a*_/*a*, σ_*a*_ being the Gaussian standard deviation of the isotropic distortion. Note that the lattice parameter *a* establishes the *surface density of the islands*, according to 

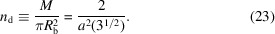

Clearly, the distance between two islands cannot be less than twice the maximum radius of the island, 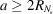 . The probability of finding an island at a distance **r**_∥_ from the first island is given by the convolution of the probability of finding an island at a distance 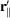 with respect to the first array along **a**_1_ and the probability of finding an island at a distance 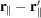 with respect to the second array along **a**_2_, 

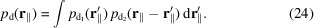

In turn, the probability 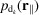 is obtained by summing the convolutions of the 2D Gaussian functions 

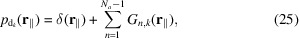




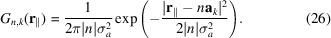

Note that the lattice vector **a**_*k*_ is given by 

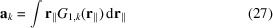

and the unique standard deviation by 

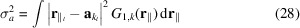

for any *k* = 1, 2 and component *l* = *x*, *y*. The island structure factor is the Fourier transform of *p*_*d*_(**r**_∥_), 

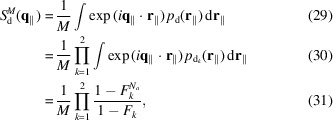




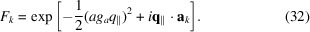




The probability of finding a pair of islands at a mutual distance **r**_∥_ is similarly obtained by the convolution of two pair probabilities, 

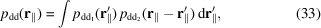

and each probability, according to PT theory, is given by a combination of convoluted Gaussians, 

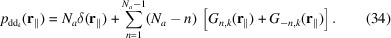

The island–island structure factor is clearly the Fourier transform of *p*_dd_(**r**_∥_), 

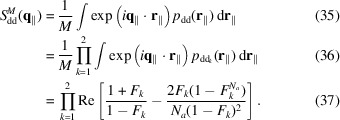




The bilayer–bilayer structure factor is calculated on the basis of one unit vector, 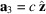 , *c* being the average stacking distance. According to Frühwirth *et al.* (2004 ▸), considering a stack of *N* bilayers with islands, the bilayer–bilayer structure factor can be written following the PT theory or the modified Caillé theory (MCT) as 

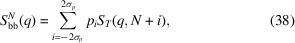

with *T* = PT or MCT, where 

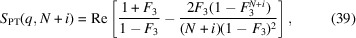




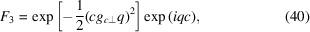




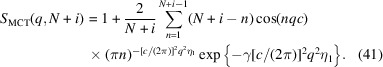


*g*_*c*⊥_ is the perpendicular distortion factor, η_1_ is the Caillé parameter and γ is Euler’s constant. According to Zhang *et al.* (1996 ▸), the distortion factor can be expressed in terms of η_1_, *g*_*c*⊥_ = (0.087η_1_)^1/2^. Polydispersity over *N* should be introduced in order to eliminate ‘intrinsic’ oscillations of the monodisperse paracrystalline structure factor at low *q* that have never been seen in experimental data (Frühwirth *et al.*, 2004 ▸). Sampling points are weighted by a discrete Gaussian distribution, as shown in equations (S1) and (S2) of the supporting information.

The differential scattering cross section per bilayer is obtained by calculating the orientational average of the squared modulus of the amplitude [equation (19)] and dividing by *N*,

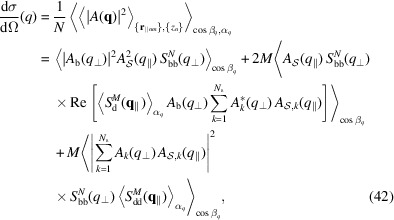

where 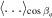 and 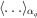 denote the zenith and azimuth averages, namely 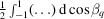 and 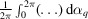 , respectively.

For a large bilayer radius (*qR*_b_ ≫ 1) the factor 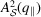 in the average integral over 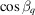 of the first term of equation (42) is dominated by asymptotic behaviour, 

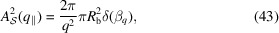

δ(β_*q*_) being the Dirac δ function. By similar arguments, it can be shown that the factor 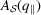 belonging to the average integral over 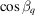 in the second term of equation (42) has the following asymptotic behaviour: 

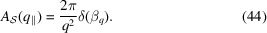

However, according to equation (23), for large *R*_b_ the number of islands *M* also becomes large and, on the basis of equation (31), the island structure factor 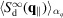 drops to zero apart from 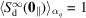 (see Fig. S1 in the supporting information). Under these conditions equation (42) reduces to 

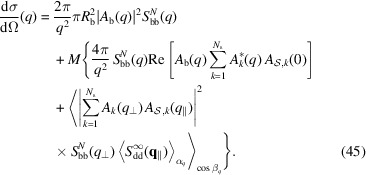




According to equation (37), the island–island structure factor for an infinite two-dimensional paracrystal is 

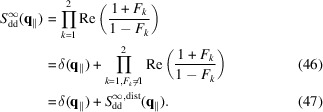




A final equation that describes the experimental scattering intensity (more properly called ‘macroscopic differential scattering cross section’) in terms of 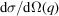 should be given by 

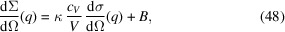

where *c*_*V*_ is the volume fraction of the whole scattering matter in the system and *V* is the volume of a single bilayer with islands. Here κ and *B* represent a scaling factor and a flat background, respectively, both due to instrumental effects. Note that, in the case of SANS, *B* could be due to incoherent neutron scattering phenomena. The volume *V* of a bilayer with islands can be expressed as a function of the thickness of a bilayer without islands, 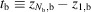 , and the thickness of the cylindrical shells, 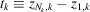 , 

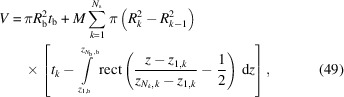




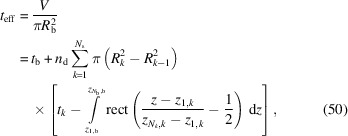

where 

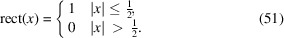




In conclusion, the scattering intensity to be fitted to the experimental data becomes a function that does not depend on *R*_b_,

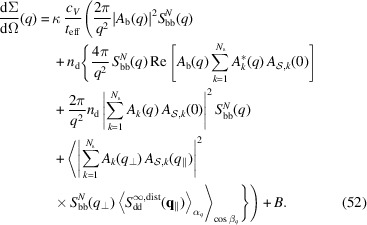




The model developed here, described by equation (52), is named SASBIN and is integrated in the *GENFIT* software (Spinozzi *et al.*, 2014 ▸). Interestingly, considering no multilayer stacking [ 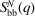 = 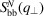 = 1] in equation (52), two limit cases may call our attention. Firstly, in the case where one has a very large island-containing bilayer, and hence the distances *a* between the islands are also very large (*n*_d_ is quite small), the resulting scattering is a mixture of two independent scatterings from the flat surfaces (bilayer and island) [see Section S2 for in-depth detail, equation (S5)]. Secondly, in the case of very small islands distributed in the lipid bilayer, the scattering intensity resolves to that of a homogeneous bilayer given by a mixture of the two electron-density profiles from the bilayer and the islands [see Section S2, equation (S10)].

In the following, we present some examples of SAXS profiles from lipid bilayers containing different scatterers, such as proteins immersed or not in the hydrophobic medium, and lipid domains with distinct SLDs with respect to the host bilayer. The question of how the presence of pores impacts on the lipid bilayer scattering will also be addressed. The corresponding SANS profiles are presented in the supporting information.

## 3. Results and discussion

### 3.1. Lipid domains

It is well known that the presence of domains in lipid bilayers can be recognized by the presence of distinct lamellar diffraction peaks over the lipid bilayer form factor measured from SAS multilamellar vesicles (Heftberger *et al.*, 2015 ▸). SANS data performed on LUVs made of a mixture of deuterated and protonated lipids of different species can give information on the protonated lipid domain size, provided the deuterated portion matches the SLD of D_2_O/H_2_O (Pencer *et al.*, 2005 ▸; Kinnun *et al.*, 2023 ▸; Heberle *et al.*, 2013 ▸). Here, we demonstrate the possibility of extracting this information from SAXS (and SANS) curves simulated according to our modelling, without the need for using SLD contrast matching.

**Figure 3:**
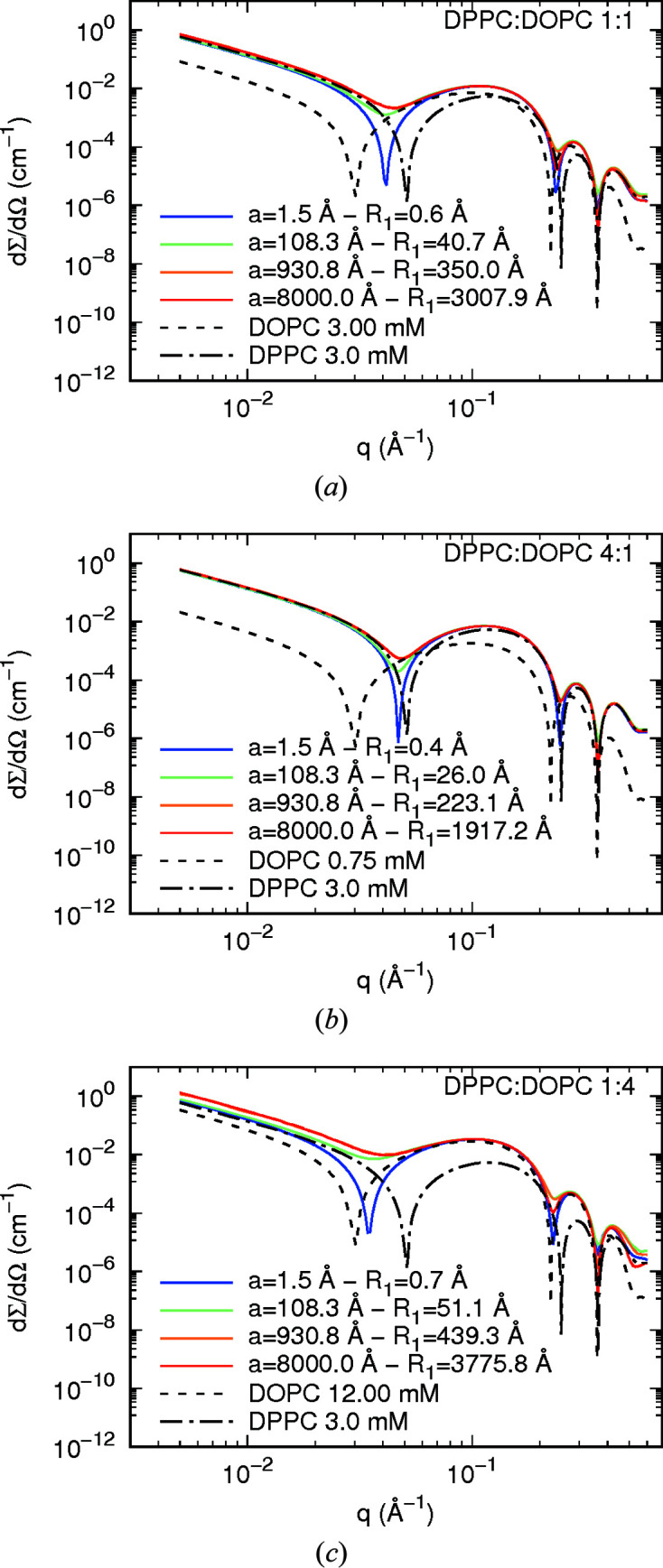
Simulated SAXS curves from coexisting DOPC fluid phase–DPPC gel phase. *C*_DPPC_ = 3 m*M* and the lattice distortion factor *g*_*a*_ = 0.3. *a* corresponds to the centre-to-centre distance between the DOPC lipid domains dispersed in the DPPC host bilayer. *R*_1_ corresponds to the radius of a cylindrical island representing a domain (Fig. S2). The surface density of domains, according to equation (23), ranges from 0.51 Å^−2^ to 1.8 × 10^−8^ Å^−2^.

Fig. 3 ▸ shows simulations of SAXS curves obtained using equation (52) (with κ = 1 and *B* = 0) of fluid–gel lipid phases formed by the coexistence of DOPC (aliphatic chains in fluid conformation) in a bilayer of DPPC (aliphatic chains in gel conformation). A schematic representation is shown in Fig. S2. Corresponding SANS curves in pure D_2_O (deuteration grade *x*_D_ = 1) are shown in Fig. S3. All calculations have been done with *C*_DPPC_ = 3 m*M*. Three distortion factors have been applied to the simulations (*g*_*a*_ = 0.1, 0.2 and 0.3). For the lower *g*_*a*_ factor, *i.e.* a low distortion factor with respect to the hexagonal array of SLDs proposed in the model, small diffraction peaks could appear over the scattering curves (Fig. S4). To be more realistic regarding SLDs distributed in a lipid bilayer system, we choose to show here all simulations with *g*_*a*_ = 0.3.

Figs. 3 ▸(*a*), 3 ▸(*b*) and 3 ▸(*c*) correspond to different DPPC:DOPC molar ratios, as indicated. In each panel, the curves refer to different sets of the lattice distance *a* and domain radius *R*_1_, which are related to the concentrations of DPPC and DOPC according to *a* ^2^ = 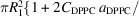 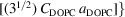 , where *a*_DPPC_ and *a*_DOPC_ are the areas per molecule in the bilayer. For both lipids, the area per molecule *a*_*l*_ (with *l* = b for DPPC and *l* = 1 for DOPC), the volume *v*_*l*_, the thicknesses *D*_*j*,*l*_, the corresponding electron densities ρ_*j*,*l*,X_ and neutron SLDs in pure D_2_O ρ_*j*,*l*,N_ (the region index *j* = 1, 2, 3 referring to the polar head region, the intermediate hydrophobic region rich in CH_2_ and the terminal region rich in CH_3_, respectively), and the smoothness parameters σ_*j*,*l*_ are shown in Table 1 ▸. They have been calculated by the best fit with equation (10) to the form factors of pure DPPC and DOPC obtained with the chemical group model (De Rosa *et al.*, 2018 ▸). The volume fraction of the sample, seen in equation (52), is *c*_*V*_ = *N*_A_(*C*_DPPC_ *v*_DPPC_ + *C*_DOPC_ *v*_DOPC_), where *N*_A_ is Avogadro’s number.

**Figure 4:**
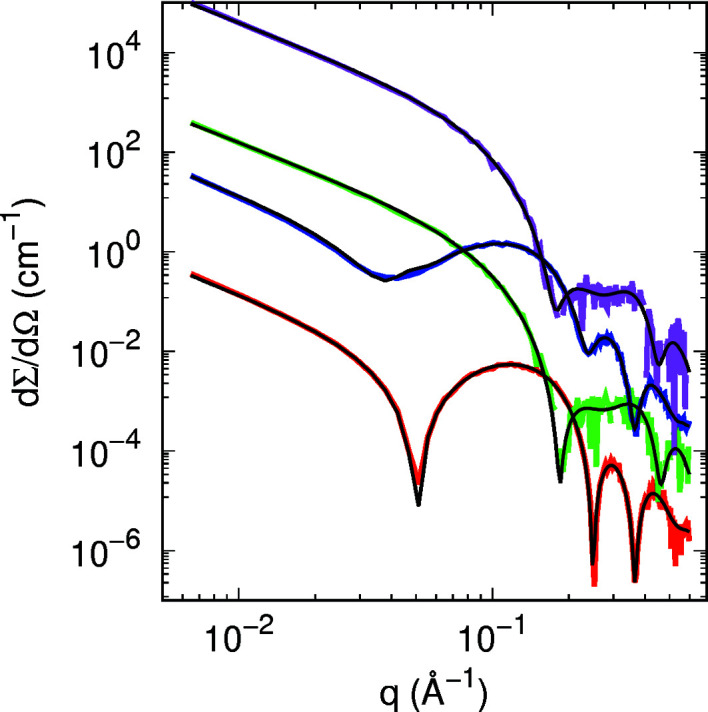
Best global fits (black lines) of simulated SAXS (red and blue lines) and SANS (green and magenta lines) curves of 3 m*M* DPPC and DOPC domains in 3 m*M* DPPC, respectively (*g*_*a*_ = 0.3, *a* = 150 Å, *R*_1_ = 60 Å, corresponding to *C*_DOPC_ = 3.94 m*M*). The simulated curves have been randomly moved by sampling from a Gaussian distribution with standard deviation proportional to [dΣ/dΩ(*q*)]^1/2^. Curves are vertically displaced for clarity.

In all panels of Fig. 3 ▸, SAXS curves of pure DOPC and pure DPPC are shown for comparison. Note that, at a DOPC:DPPC molar ratio of 1:1 [Fig. 3 ▸(*a*)], the resulting scattering has the minima of the form factor as the mean *q* positions between the minima of the DPPC and DOPC scattering curves. These minima are displaced towards the minima of the DPPC or DOPC SAXS curves according to the increase in the amount of DPPC or DOPC in the lipid bilayer, respectively [Figs. 3 ▸(*b*) and 3 ▸(*c*)]. Interestingly, for a small DOPC domain size (*a* = 1.5 Å) one can clearly observe a deep first minimum arising from the mixture of the two electron-density profiles, taking into account the surface fraction of each component [DOPC domains and the DPPC bilayer, equation (S10) of the supporting information]. On the other hand, for large DOPC domains, the minima become very shallow with respect to those observed from small domains, reflecting the fact that the scattering of two independent scatterers is also weighted by the surface fraction of each component [equation (S5) of the supporting information]. It is evident that the size of the domain, *i.e.* the radius *R*_1_ of the island representing an SLD inhomogeneity, can be retrieved from the SAXS curve since the depth of the minima of the oscillations is related to the parameter *a* and hence to *R*_1_ [see, for instance, the result presented for *a* = 108.3 Å, green lines in Figs. 3 ▸(*a*)–3(*c*)]. The scattering intensities dΣ/dΩ(*q*) also depend on the *a* (and *R*_1_) values, since they are related to the surface density of the islands *n*_d_ according to equation (52). The SANS profiles display significant differences on changing the *a* parameter and DOPC:DPPC molar ratio (Fig. S3). As a consequence, a combined SAXS/SANS analysis can give strong support to the structural parameters obtained from lipid-domain-containing bilayers. As an example, we present the consistency of the combined SAXS/SANS data analysis performed by the *GENFIT* software from a lipid bilayer composed of DOPC:DPPC in a 1:1 molar ratio, with input parameters *a* = 150 Å, *R*_1_ = 60 Å and *g*_*a*_ = 0.3 (see Fig. 4 ▸ and Table 2 ▸). Note that the output parameters are quite similar to the ones considered as input parameters.

### 3.2. Proteins-containing lipid bilayers

**Figure 5:**
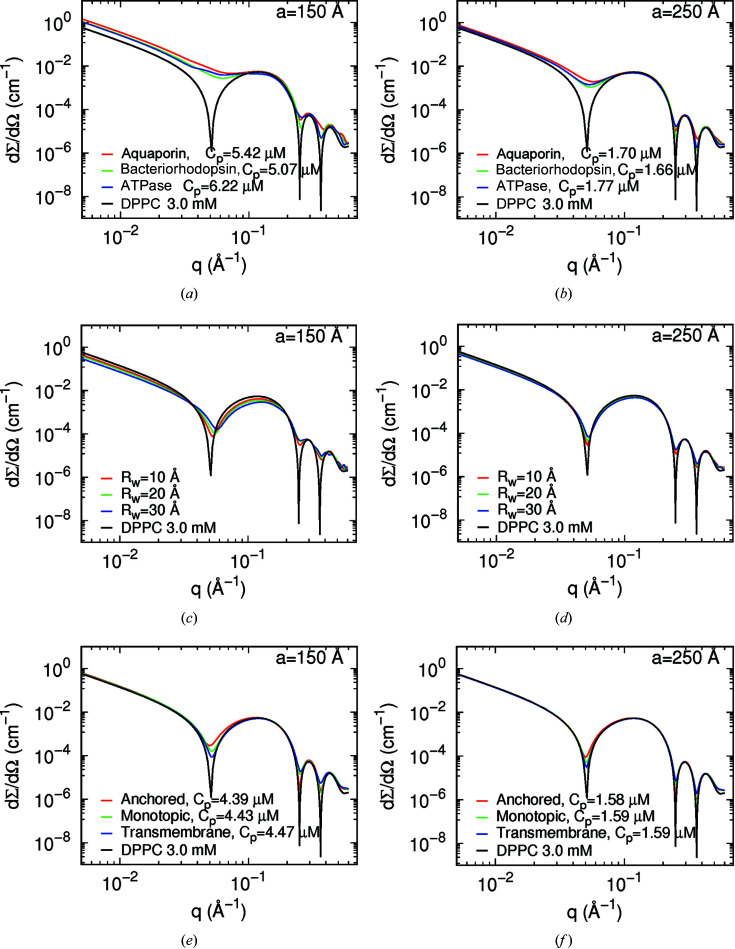
Simulated SAXS curves of islands in a DPPC bilayer at *C*_DPPC_ = 3 m*M* according to the SASBIN model. (*a*) and (*b*) Three different transmembrane proteins (as indicated) with lattice parameter *a* of (*a*) 150 Å and (*b*) 250 Å. (*c*) and (*d*) Three different water pores (with indicated pore radius *R*_w_) with lattice parameter *a* of (*c*) 150 Å and (*d*) 250 Å. (*e*) and (*f*) Three different positions of cytochrome c (as indicated) with lattice parameter *a* of (*e*) 150 Å and (*f*) 250 Å. In all cases the distortion parameter is *g*_*a*_ = 0.3.

Fig. 5 ▸ shows the simulated SAXS curves obtained with *a* = 150 Å (left-hand panels) and *a* = 250 Å (right-hand panels) for three transmembrane proteins [panels (*a*) and (*b*)], for water pores [panels (*c*) and (*d*)] and for cytochrome c in different positions with respect to the bilayer centre [panels (*e*) and (*f*)]. Such systems are represented in Figs. S5–S8. In all cases, the simulations have been done at 3 m*M* DPPC and with a unique island–island distortion factor *g*_*a*_ = 0.3. Corresponding SANS simulations at *x*_D_ = 1 are shown in Fig. S9.

**Figure 6:**
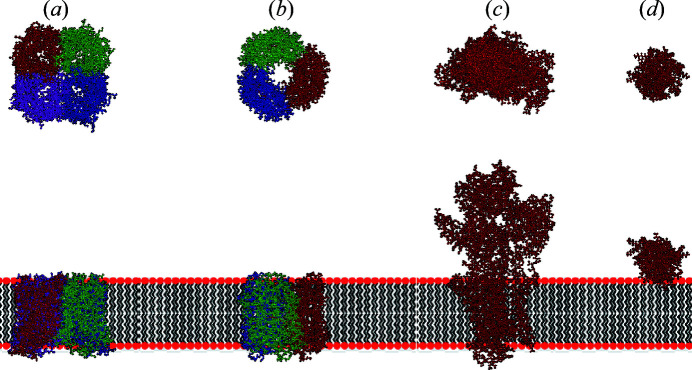
Ribbon representations of (*a*) aquaporin (PDB code 3d9s; Horsefield *et al.*, 2008 ▸), (*b*) bacteriorhodopsin (PDB code 1fbb; Subramaniam & Henderson, 2000 ▸), (*c*) ATPase (PDB code 3wgv; Kanai *et al.*, 2003 ▸) and (*d*) cytochrome c (PDB code 1giw; Banci *et al.*, 1999 ▸). (Top row) Views in the *xy* plane of the membrane. (Bottom row) Views across the membrane (*xz* plane) together with a cartoon representation of the lipid bilayer.

The form factors of each protein (represented in Fig. 6 ▸) were determined with the SASMOL method (Ortore *et al.*, 2009 ▸) without considering the difference in the mass density between the first hydration shell water and bulk water. Subsequently, the calculated form factors were fitted with a core–shell cylinder model, according to equation (S11) shown in the supporting information. Note that this approximation is necessary in order subsequently to describe the proteins embedded in the membranes using the island model introduced in Section 2. The fitting parameters are the radius *R* of the cylinder core, the shell thickness δ, and the fractions ϕ_i_ and ϕ_e_ of polypeptide matter in the core and shell regions, respectively, described in Table 3 ▸. Curves simulated with SASMOL and the best fits obtained by the core–shell cylinder model are shown in Fig. S10. The fitted parameters were related to the cylindrical islands used to describe the structures of the transmembrane proteins (aquaporin, bacteriorhodopsin and ATPase) in a DPPC bilayer with the SASBIN model. In detail, the island is formed by two cylindrical shells (*N*_s_ = 2), with *N*_1_ = 1 and *N*_2_ = 1. The corresponding radii are *R*_1_ = *R* and *R*_2_ = *R* + δ and the SLDs are those obtained by the core–shell cylinder model [equations (S13) and (S14) of the supporting information]. The thicknesses are *D*_1,1_ = *D*_1,2_ = *L*. For aquaporin and bacteriorhodopsin, to simulate perfect positioning within the bilayer, we set *z*_1,1_ = *z*_1,2_ = −*L*/2. On the other hand, for ATPase, since almost half of the protein’s longest dimension is embedded in the bilayer [see Fig. 6 ▸(*c*)], we set *z*_1,1_ = *z*_1,2_ = −*L*/4. Considering the area of the protein towards the *xy* plane, 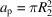 , and the area per polar head of DPPC, *a*_DPPC_, an expression of the protein concentration *C*_p_ as a function of the lattice parameter *a* can be easily derived, *C*_p_ = *C*_b_ *a*_DPPC_/[(3^1/2^)*a* ^2^ − 2*a*_p_]. Values are reported in the legends of Fig. 5 ▸(*a*) and 5 ▸(*b*). The volume fraction of the sample is *c*_*V*_ = *N*_A_(*C*_b_ *v*_DPPC_ + *C*_p_ *v*_p_).

**Figure 7:**
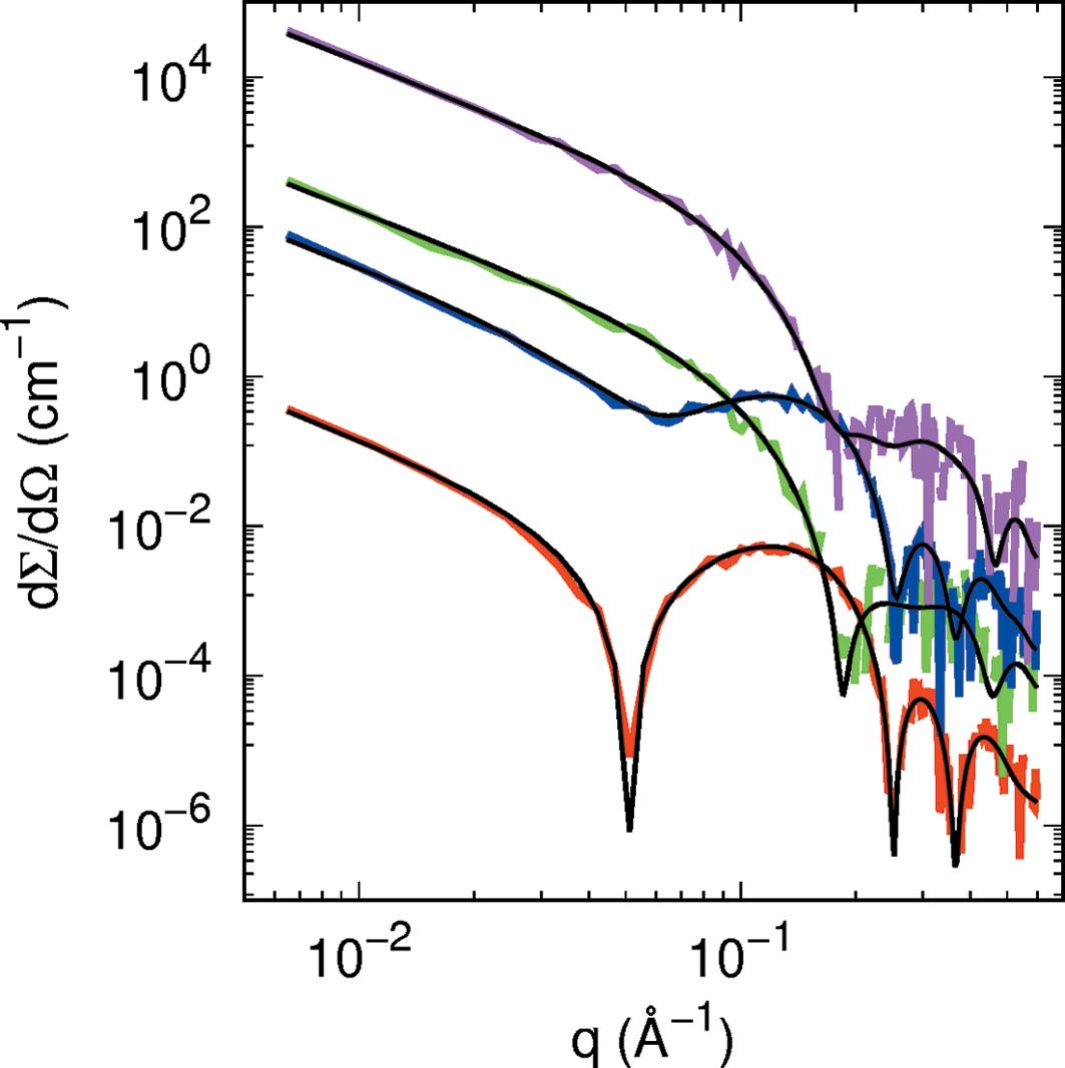
Best global fits (black lines) of SAXS (red and blue lines) and SANS (green and magenta lines) curves of DPPC and bacteriorhodopsin in DPPC according to the simulations shown in Fig. 5 ▸(*a*) and Fig. S9(*a*). The simulated curves have been randomly moved by sampling from a Gaussian distribution with standard deviation proportional to [dΣ/dΩ(*q*)]^1/2^. Curves are vertically displaced for clarity.

It should be noted that *a* values of 150 and 250 Å were chosen because the largest studied protein radius (ATPase) is 42.8 Å, *i.e.* a total cylindrical diameter of *ca* 86 Å. Thus, the distance between the islands was less than twice or three times their diameter, for comparison. Furthermore, each of the transmembrane proteins has an aqueous pore of different dimension ranging from 5.8 to 18 Å (Table 3 ▸). Although the proteins produce different scattering profiles with respect to the protein-free DPPC bilayer [Figs. 5 ▸(*a*) and 5 ▸(*b*)], the most marked effect is the smoothness of the oscillation minima, which is correlated to the *a* values as discussed for lipid domains. Note that the ratio between the first, second and third minimum depths can vary depending on the protein species. Moreover, the scattering intensity varies with SLD contrast between the island and the surrounding medium. In the case of SANS profiles (Fig. S9), all investigated protein profiles are quite distinguishable for *q* values between 0.2 and 0.4 Å^−1^. Therefore, the combined SAXS/SANS experiments allow us to obtain information about the dimensions of the immersed proteins in the lipid bilayer. For the sake of validation, Fig. 7 ▸ shows the simulated SAXS and SANS experiments for bacteriorhodopsin, along with the best fitting result obtained with *GENFIT*, while Table 4 ▸ displays the input and output fitting parameters. The good agreement between them confirms the robustness of the SASBIN method. For the sake of completeness, further *GENFIT* analyses of simulated SAXS/SANS curves regarding aquaporin and ATPase in a DPPC bilayer are reported in Figs. S11 and S12, respectively, and the fitting parameters are shown in Tables S1 and S2.

The case of cytochrome c, here simply considered an example of a model protein that could interact differently with a membrane in an anchored (ξ = 0), a monotopic (ξ = 1/2) or a transmembrane geometry (ξ = 1), is different. For an anchored configuration, we have, for both shells (*N*_s_ = 2, with radii *R*_1_ = *R* and *R*_2_ = *R* + δ), six levels (*N*_1_ = *N*_2_ = 6), the first five levels being equal to those of the bilayer and the sixth level constituted by the protein. For a monotopic configuration, for both shells (*N*_s_ = 2, with radii *R*_1_ = *R* and *R*_2_ = *R* + δ) we have four levels, the first three levels being equal to those of the bilayer and the fourth level constituted by the protein. The protein concentration is related to the lattice parameter by *C*_p_ = *C*_b_ *a*_DPPC_/[(3^1/2^)*a* ^2^ − 2ξ*a*_p_]. Simulated SAXS and SANS curves for the three cases are shown in Figs. 5 ▸(*e*) and 5 ▸(*f*) and Figs. S9(*e*) and S9(*f*), respectively.

**Figure 8:**
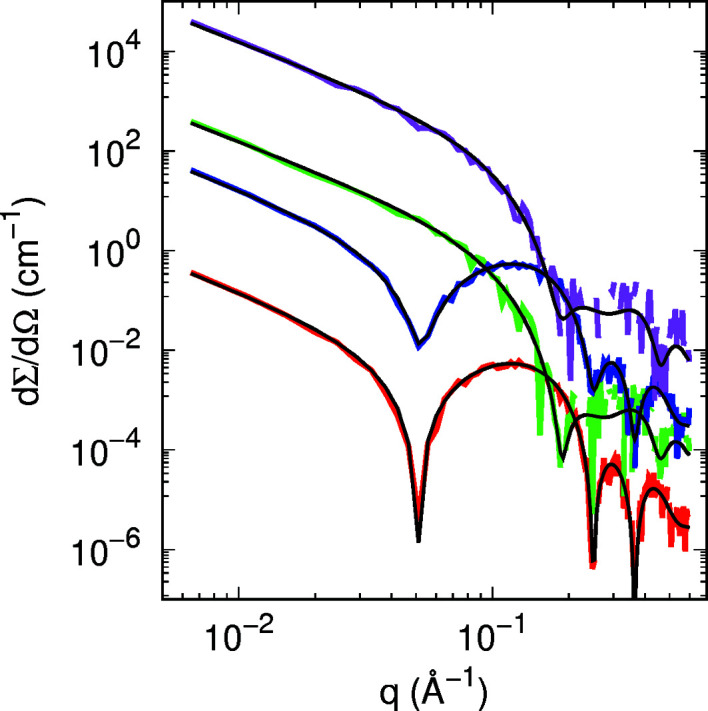
Best global fits (black lines) of SAXS (red and blue lines) and SANS (green and magenta lines) curves of DPPC and cytochrome c in DPPC in the monotopic configuration according to the simulations shown in Fig. 5 ▸(*e*) and Fig. S9(*e*). The simulated curves have been randomly moved by sampling from a Gaussian distribution with standard deviation proportional to [dΣ/dΩ(*q*)]^1/2^. Curves are vertically displaced for clarity.

As one can see from Figs. 5 ▸(*e*) and 5(*f*), the signature of anchored, monotopic and transmembrane proteins can also be verified through the ratio between the minimum depths from SAXS curves, whereas differences in the SANS profiles with respect to the cytochrome c free DPPC membrane mainly occurs at *q* ranging from 0.2 to 0.4 Å^−1^. Once again, one can retrieve significant information about anchoring or intercalation of the protein in the lipid membrane from combined SAXS/SANS data analysis. As an example, Fig. 8 ▸ presents the simulated experiment along with the best fitting result, and Table 5 ▸ displays the input and output fitting parameters, for cytochrome c in a monotopic configuration, demonstrating the completeness of the methodology used here.

### 3.3. Pore-containing membranes

Water pores with radius *R*_w_ are described by the geometry of the inner surface of a torus (Spinozzi *et al.*, 2010 ▸). A representation is provided in Fig. S6. This geometry is mapped onto a three-level cylinder island (*N*_s_ = 3), with radii *R*_1_ = *R*_w_, *R*_2_ = *R*_w_ + *D*_1,b_ and *R*_3_ = *R*_w_ + *D*_1,b_ + *D*_2,b_ + *D*_3,b_, and the corresponding number of SLD levels *N*_1_ = 0, *N*_2_ = 1 and *N*_3_ = 3. The lower *z* level of the first shell *z*_1,2_ and the thickness of the region *D*_1,2_ are analytically calculated in such a way that the volume of the cylindrical shell is equal to the volume of the part of the inner torus with an *x* projection between −*R*_2_ and −*R*_1_, as shown in Fig. S6. The lower *z* level of the second shell *z*_1,3_ and the thicknesses of the two regions *D*_1,2_ and *D*_2,2_ are calculated in such a way that the volumes of the two cylindrical shells are equal to the volumes of the two parts of the inner torus with an *x* projection between −*R*_3_ and −*R*_2_, as shown in Fig. S6. The results lead to these expressions: *z*_1,1_ = −*F*_6_ *D*_1,b_, *D*_1,1_ = 2*F*_6_ *D*_1,b_, *z*_1,2_ = −*F*_7_ *D*_1,*b*_ + *F*_4_ *F*_1_, *D*_1,2_ = *F*_7_ *D*_1,*b*_ and *D*_2,2_ = 2*F*_4_ *F*_1_. The factors *F*_1_ to *F*_7_ are given in Section S4. Note that they depend on the pore radius *R*_w_. Simulated SAXS and SANS curves for the three values of *R*_w_ are shown in Figs. 5 ▸(*c*) and 5 ▸(*d*) and in Figs. S9(*c*) and S9(*d*), respectively.

As one can see from Figs. 5 ▸(*c*) and 5 ▸(*d*), it is possible to recognize different pore dimensions, ranging from 10 to 30 Å, from the SAXS curves since the scattering profiles may have different minimum *q* positions and the intensities are smaller than those produced by a pore-free bilayer, due to differences in the SLD contrast between the pore and the bilayer. Concomitantly, the SANS curves also reflect the scattering differences of membranes containing pores [Figs. S9(*c*) and S9(*d*)]. Therefore, the combined SANS/SAXS results can furnish the values of the pore radius *R*_w_, such as those produced for instance by peptides and toxins interacting with membranes, and of the numerical density *n*_d_ when SLD inhomogeneities are accounted for as here proposed by the SASBIN model.

## 4. Concluding remarks

Small-angle X-ray and neutron scattering have traditionally been used to determine the structure of single-component biomimetic membranes that can be represented by unilamellar and multilamellar vesicles. SAXS and SANS intensities over a *q* range are intrinsically related to the Fourier transform of the SLD profile of the lipid bilayer. In the simplest analysis commonly reported in the literature, the lipid bilayer thickness and the SLDs of both polar and hydrophobic regions are considered free parameters that can be determined by the best fit to the experimental SAS curve. More recently, a method considering the scattering of lipid chemical groups has been introduced in the literature (Wiener & White, 1991*a* ▸,*b* ▸; Pan *et al.*, 2015 ▸, 2012 ▸; De Rosa *et al.*, 2018 ▸) which allows us to extract more in-depth structural details of membranes composed of a mixture of lipids. SANS has also been applied to investigating the size of lipid domains by contrast matching (Pencer *et al.*, 2005 ▸; Heberle *et al.*, 2013 ▸) and proteins inserted into the membrane (Spinozzi *et al.*, 2022 ▸). In this work, we have presented the new SASBIN model to analyse SAXS and SANS curves from large uni­lamellar vesicles containing SLD inhomogeneities. These may represent pores and lipid domains distributed in the lipid bilayer, or proteins anchored or immersed in the membrane. Through development of the SASBIN model, we have shown it is possible to recognize the presence of inhomogeneities in the SAS curves, and their dimensions and spatial distribution.

## 5. Related literature

For further literature related to the supporting information, see Jacrot (1976 ▸).

## Supplementary Material

Additional derivations, figures and tables. DOI: 10.1107/S1600576723006143/ge5136sup1.pdf

## Figures and Tables

**Table 1:** DPPC and DOPC parameters used in the simulations

Parameter	DPPC (*l* = b)	DOPC (*l* = 1)
*a*_*l*_ (Å^2^)	57	60
*v*_*l*_ (Å^3^)	1140	1270
*D*_1,*l*_ (Å)	12.0	11.9
*D*_2,*l*_ (Å)	10.7	9.9
*D*_3,*l*_ (Å)	3.50	5.87
ρ_1,*l*,X_ (e Å^−3^)	0.412	0.408
ρ_2,*l*,X_ (e Å^−3^)	0.317	0.302
ρ_3,*l*,X_ (e Å^−3^)	0.245	0.264
ρ_1,*l*,N_ (10^−6^ Å^−2^)	4.203	4.294
ρ_2,*l*,N_ (10^−6^ Å^−2^)	−0.349	−0.265
ρ_3,*l*,N_ (10^−6^ Å^−2^)	−0.602	−0.446
σ_1,*l*_ (Å)	2.49	3.50
σ_2,*l*_ (Å)	2.39	3.48
σ_3,*l*_ (Å)	2.01	4.02

**Table 2:** Parameters from the best fit to the SAS curves shown in Fig. 4 ▸ representing DOPC domains in a DPPC bilayer The length unit is ångströms. For X-rays, electron densities are expressed in e Å^−3^. For neutrons, SLDs are expressed in 10^−6^ Å^−2^.

Parameter	In	Out
*R*_1_	60.0	59 ± 1
*D*_1,b_	12.0	12.01 ± 0.03
*D*_2,b_	10.6	10.78 ± 0.03
*D*_3,b_	3.64	3.45 ± 0.04
ρ_1,b,X_	0.411	0.4123 ± 0.0002
ρ_1,b,N_	4.29	4.22 ± 0.03
ρ_2,b,X_	0.316	0.3163 ± 0.0001
ρ_2,b,N_	−0.618	−0.76 ± 0.02
ρ_3,b,X_	0.247	0.2427 ± 0.0006
ρ_3, b, N_	−0.126	−0.15 ± 0.07
σ_1,b_	2.56	2.71 ± 0.04
σ_2,b_	2.22	2.30 ± 0.04
σ_3,b_	1.74	2.05 ± 0.06
*D*_1,1_	12.3	12.47 ± 0.06
*D*_2,1_	12.3	11.60 ± 0.05
*D*_3,1_	3.13	3.74 ± 0.03
ρ_1,1,X_	0.407	0.4054 ± 0.0003
ρ_1,1,N_	4.33	4.19 ± 0.04
ρ_2,1,X_	0.296	0.2965 ± 0.0002
ρ_2,1,N_	−0.529	−0.59 ± 0.08
ρ_3,1,X_	0.246	0.2578 ± 0.0008
ρ_3,1,N_	−0.106	−0.21 ± 0.07
σ_1,1_	3.47	3.40 ± 0.07
σ_2,1_	3.51	3.24 ± 0.09
σ_3,1_	4.12	3.0 ± 0.2
*g*_*a*_	0.300	0.197 ± 0.002

**Table 3:** Protein parameters used in the simulations of SAXS and SANS curves shown in Fig. S10

	Aquaporin (PDB 3d9s)	Bacteriorhodopsin (PDB 1fbb)	ATPase (PDB 3wgv)	Cytochrome c (PDB 1giw)
*M*_w_ (g mol^−1^)	102 505	73 690	109 942	11 721
*V*_p_ (Å^3^)	133 006	96 506	138 965	14 839
*R* (Å)	5.78	8.01	18.2	0.262
δ (Å)	28.6	21.0	24.6	15.0
ϕ_i_	0.211	0.0195	1.00	0.718
ϕ_e_	0.904	0.870	0.0557	0.886
*L* † (Å)	41.4	45.5	107	22.8
ρ_p,X_ (e Å^−3^)	0.411	0.411	0.420	0.420
ρ_p,N_ (10^−6^ Å^−2^)	2.828	2.732	3.009	3.119

† Input parameters are derived parameters.

**Table 4:** Best fit parameters of the curves shown in Fig. 7 ▸ representing the transmembrane protein bacteriorhodopsin in a DPPC bilayer The length unit is ångströms. For X-rays, electron densities are expressed in e Å^−3^. For neutrons, SLDs are expressed in 10^−6^ Å^−2^.

	In	Out
*R*_1_	8.01	8 ± 1
*t*	21.0	20.8 ± 0.6
ϕ_e_	0.870	0.800 ± 0.003
ϕ_i_	0.0195	0.05 ± 0.02
*L* †	45.5	49.2 ± 0.7
*D*_1,b_	12.0	11.6 ± 0.2
*D*_2,b_	10.6	11.3 ± 0.1
*D*_3,b_	3.64	3.0 ± 0.1
ρ_1,b,X_	0.411	0.414 ± 0.001
ρ_1,b,N_	4.29	4.26 ± 0.05
ρ_2,b,X_	0.316	0.318 ± 0.001
ρ_2,b,N_	−0.618	−0.80 ± 0.06
ρ_3,b,X_	0.247	0.231 ± 0.002
ρ_3,b,N_	−0.126	−0.2 ± 0.3
σ_1,b_	2.56	2.9 ± 0.2
σ_2,b_	2.22	2.34 ± 0.07
σ_3,b_	1.74	2.4 ± 0.2
*g*_*a*_	0.300	0.36 ± 0.02

† Input parameters are derived parameters.

**Table 5:** Best fit parameters of curves shown in Fig. 8 ▸ representing cytochrome c in a DPPC bilayer with a monotopic configuration The length unit is ångströms. For X-rays, electron densities are expressed in e Å^−3^. For neutrons, SLDs are expressed in 10^−6^ Å^−2^.

	In	Out
*R*_1_	0.262	1.9 ± 0.7
δ	15.0	12 ± 1
ϕ_e_	0.886	1.0 ± 0.1
ϕ_i_	0.718	1.0 ± 0.2
*L* †	22.8	22 ± 1
ξ	0.500	0.7 ± 0.2
*D*_1,b_	12.0	11.9 ± 0.1
*D*_2,b_	10.6	11.07 ± 0.06
*D*_3,b_	3.64	3.12 ± 0.04
ρ_1,b,X_	0.411	0.414 ± 0.001
ρ_1,b,N_	4.29	4.25 ± 0.07
ρ_2,b,X_	0.316	0.3143 ± 0.0008
ρ_2,b,N_	−0.618	−0.61 ± 0.08
ρ_3,b,X_	0.247	0.241 ± 0.001
ρ_3,b,N_	−0.126	−0.3 ± 0.2
σ_1,b_	2.56	2.69 ± 0.08
σ_2,*b*_	2.22	2.5 ± 0.1
σ_3,b_	1.74	1.6 ± 0.1
*g*_*a*_	0.300	0.42 ± 0.03

† Input parameters are derived parameters.

## Footnotes

1 Lipid abbreviations used are as follows: DOPC is dioleoylphosphatidylcholine, DPPC is dipalmitoylphosphatidylcholine, POPC is 1-palmitoyl-2-oleoyl­phosphatidylcholine, DSPC is distearoylphosphatidyl­choline and DLPC is dilauroylphosphatidylcholine.
