# Supplementary material for: Small-angle scattering from flat bilayers containing correlated scattering length density inhomogeneities
Source: J Appl Crystallogr. 2023 Aug 16;56(Pt 5):1348–60. doi: 10.1107/S1600576723006143 (PMC10543680; doi:10.1107/S1600576723006143)
Supplement: Supplementary file 1 [file j-56-01348-sup1.pdf]

JOURNAL OF
APPLIED
CRYSTALLOGRAPHY

Volume 56 (2023)

Supporting information for article:

Small-angle scattering from flat bilayers containing correlated scattering length density inhomogeneities

Francesco Spinozzi, Leandro R. S. Barbosa, Giacomo Corucci, Paolo Mariani and Rosangela Itri

Supporting information

Small-angle scattering from flat bilayers containing correlated scattering length density inhomogeneities

Francesco Spinozzi[a], Leandro R. S. Barbosa[b,c], Giacomo Corucci[d,e] , Paolo Mariani[a] and Rosangela Itri[b]

[a] Department of Life and Environmental Sciences, Marche Polytechnic University, Ancona, Italy

[b] Institute of Physics, University of São Paulo, São Paulo, Brazil

[c] Brazilian Synchrotron Light Laboratory (LNLS), Campinas, São Paulo, Brazil

S1 Discrete Gaussian distribution of polydisperse paracrystalline order

$$p_i \;=\; \frac{e^{-\frac{1}{2}i^2/\sigma_p}}{\sum_{i=-2\sigma_p}^{2\sigma_p} e^{-\frac{1}{2}i^2/\sigma_p^2}} \qquad \text{(S1)}$$

with standard deviation

$$\sigma_p = \begin{cases} \frac{1}{2}(N-1) & N < 5 \\ N^{1/2} & N \geq 5 \end{cases} \qquad \text{(S2)}$$

S2 Limit cases

Let us suppose to have islands formed by only one cylindrical shell ($N_s = 1$), with a finite radius R_1, without any vertical correlation ($S_{bb}^N(q) = 1$). We imagine that the radius R_1 of the unique shell is very large so that in the average integral over $\cos\beta_q$ of the third term we have $A_{\mathcal{S},1}^2(q) = \frac{2\pi}{q^2}\pi R_1^2 \delta(\beta_q)$ and, obviously, $A_{\mathcal{S},1}(0) = \pi R_1^2$. The distance a among islands will also be very big so that we can assume that the island-island structure factor $<S_{dd}^{\infty}(\mathbf{q}_{\|})>_{\alpha_q}$ is always 1. Eq. 52 transforms into

$$\frac{d\Sigma}{d\Omega}(q) \;=\; \kappa \frac{c_V}{t_{\text{eff}}} \frac{2\pi}{q^2} \left\{ |A_b(q)|^2 + n_d \pi R_1^2 \left[2\text{Re}\left\{ A_b(q) A_1^*(q) \right\} + |A_1(q)|^2 \right] \right\} + B \qquad \text{(S3)}$$

Now, we recognize that $n_d \pi R_1^2$ is the surface fraction of the islands, which we call c_{Sd}. Since, by definition $A_1(q) = A_d(q) - A_b(q)$, where we have defined

$$A_d(q) \quad = \quad \int dz\, [\rho_1(z) - \rho_0] e^{iqz}, \tag{S4}$$

by substituting, we arrive to the following equation

$$\frac{d\Sigma}{d\Omega}(q) \quad = \quad \kappa \frac{c_V}{t_{\text{eff}}} \frac{2\pi}{q^2} \left\{ c_{Sb} |A_b(q)|^2 + c_{Sd} |A_d(q)|^2 \right\} + B \tag{S5}$$

where $c_{Sb} = 1 - c_{Sd}$ is the surface fraction of the flat bilayer. Clearly, this is the mixture of two independent signal of flat surfaces.

We secondly imagine to have islands very small, so that both R_1 and a approach a limit zero value. If islands are totally uncorrelated, any distance among them has the same probability. In these conditions, the the island-island structure factor (see Eq. 19) become

$$S_{dd}^M(\mathbf{q}_\parallel) \quad = \quad \frac{1}{M} < \sum_{m,m'=1}^{M} e^{i\mathbf{q}_\parallel \cdot \mathbf{r}_{\parallel nn'mm'}} >_{\mathbf{r}_{\parallel nn'mm'}} \tag{S6}$$

$$= \quad \frac{1}{M} \sum_{m,m'=1}^{M} \frac{\int s_b(\mathbf{r}_\parallel) e^{i\mathbf{q}_\parallel \cdot \mathbf{r}_\parallel} d\mathbf{r}_\parallel}{\int s_b(\mathbf{r}_\parallel) d\mathbf{r}_\parallel} \tag{S7}$$

$$= \quad \frac{M}{\pi R_b^2} A_{\mathcal{S}}(q_\parallel) \tag{S8}$$

For large value of R_b, according to Eq 44, in the integral over $\cos\beta_q$ of the third term we obtain $S_{dd}^M(\mathbf{q}_\parallel) = n_d \frac{2\pi}{q^2} \delta(\beta_q)$. By substituting in the Eq. 52 we obtain

$$\frac{d\Sigma}{d\Omega}(q) \quad = \quad \kappa \frac{c_V}{t_{\text{eff}}} \frac{2\pi}{q^2} \left\{ |A_b(q)|^2 + n_d \left[2\pi R_1^2 \operatorname{Re} \left\{ A_b(q) A_1^*(q) \right\} + n_d (\pi R_1^2)^2 \left| A_1(q) \right|^2 \right] \right\} + B \tag{S9}$$

and, after some algebraic transformations, we arrive to the following expression

$$\frac{d\Sigma}{d\Omega}(q) \quad = \quad \kappa \frac{c_V}{t_{\text{eff}}} \frac{2\pi}{q^2} \left| c_{Sb} A_b(q) + c_{Sd} A_d(q) \right|^2 + B \tag{S10}$$

If is evident that this latter equation represents the scattering intensity of a homogeneous bilayer obtained by mixing two electron density profiles $\rho_b(z)$ and $\rho_d(z)$.

S3 Form factor of a core-shell cylinder

The form factor of a core-shell cylinder with inner radius R, shell thickness δ and length L reads

$$
\begin{aligned}
P(q) \quad = \quad & \int_0^{\pi/2} d\beta \sin\beta \left\{ 2\pi L \frac{\sin\left(\frac{1}{2}qL\cos\beta\right)}{\frac{1}{2}qL\cos\beta} \right. \\
& \times \left[(\rho_e - \rho_0)(R+\delta)^2 \frac{J_1(q(R+\delta)\sin\beta)}{q(R+\delta)\sin\beta} \right. \\
& \times \left. \left. +(\rho_i - \rho_e)R^2 \frac{J_1(qR\sin\beta)}{qR\sin\beta} \right] \right\}^2
\end{aligned} \tag{S11}
$$

where ρ_0 is the solvent SLD, ρ_i and ρ_e are the core and the shell SLD. To take into account the protein volume constraint V_p that can be calculated on the basis of the protein primary sequence[1], the following constraints have been introduced

$$V_p = \pi L\{\phi_i R^2 + \phi_e[(R+\delta)^2 - R^2]\} \tag{S12}$$

$$\rho_i = \phi_i \rho_p + (1-\phi_i)\rho_0 \tag{S13}$$

$$\rho_e = \phi_e \rho_p + (1-\phi_e)\rho_0 \tag{S14}$$

where ϕ_i and ϕ_e represent the volume fraction of polypeptides in the core and the shell, ρ_p is the protein SLD calculated on the basis of the primary sequence and ρ_0 is the solvent SLD.

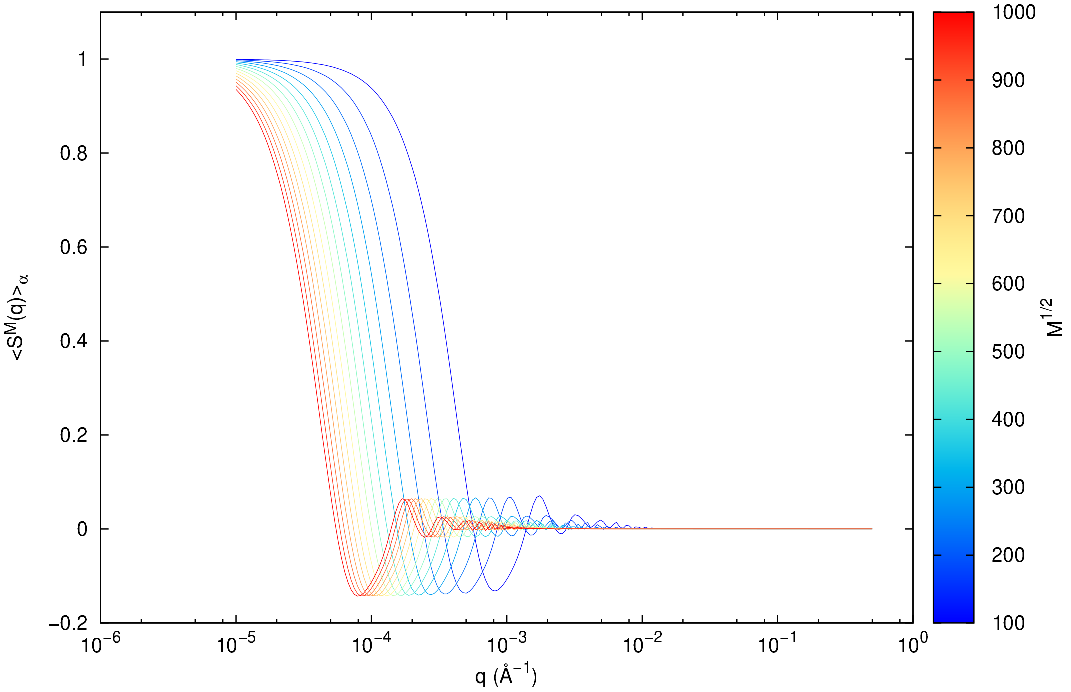

Figure S1: Plot of the function $<S_d^M(\mathbf{q}_{\|})>_{\alpha_q}$ for different values of M, as indicated in the palette.

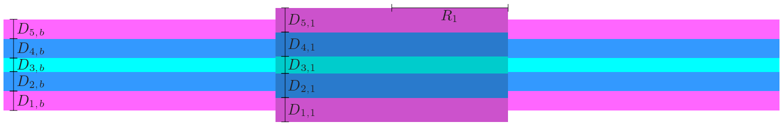

Figure S2: Lipid domain in a membrane.

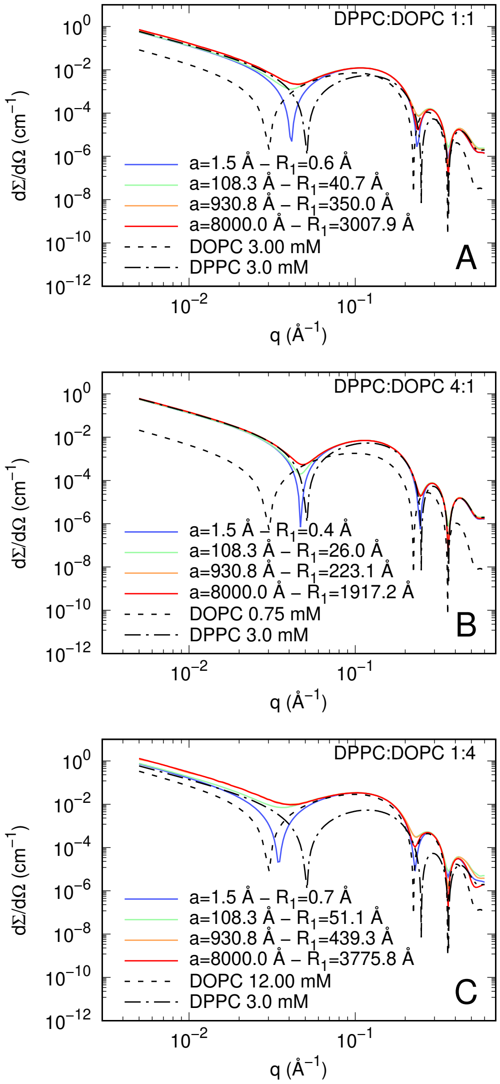

Figure S3: Simulated SANS curves at $x_D = 1$ from DOPC fluid-DPPC gel phase coexistence. $C_{\text{DPPC}} = 3$ mM and the lattice distortion factor $g_a = 0.3$. a corresponds to the center to center distance among the DOPC lipid domains dispersed in DPPC host bilayer. R_1 corresponds to the radius of a cylindrical island representing the domain (see Fig. S2 of the SI). The surface density of domains, according to Eq. 23, ranges from 0.51 Å^{-2} to $1.8 \cdot 10^{-8}$ Å^{-2}.

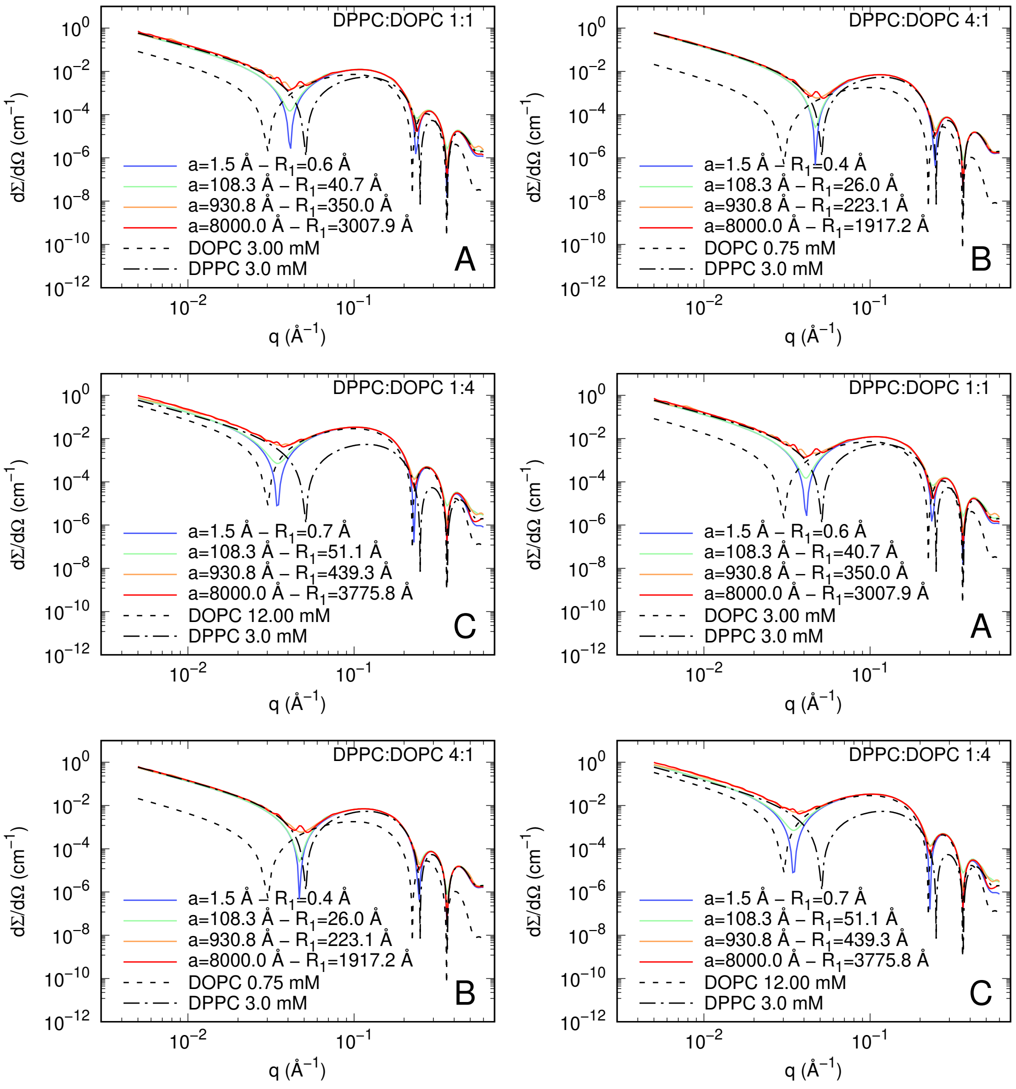

Figure S4: Simulated SAXS curves (panels A, B and C) and SANS curves at $x_D = 1$ (panels D, E and F) from DOPC fluid-DPPC gel phase coexistence. $C_{\mathrm{DPPC}} = 3$ mM and the lattice distortion factor $g_a = 0.1$.

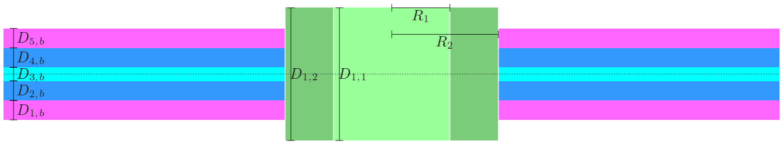

Figure S5: Transmembrane protein.

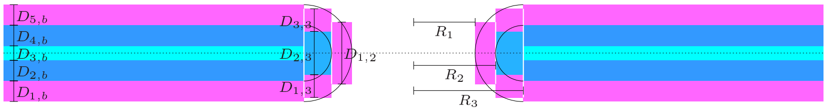

Figure S6: Representation of a water pore approximated with a three-level cylindrical island.

S4 Factors to approximate the toroidal geometry of a pore with a three-level cylindrical island

$F_1 = D_{2,b} + D_{3,b}$

$F_2 = (R_w + D_{1,b} + F_1)$

$F_3 = \sqrt{1 - F_1^2/(D_{1,b} + F_1)^2}$

$F_4 = (4F_1 - 3\pi F_2)/(6F_1 - 12F_2)$

$F_5 = \cos^{-1}(F_1/(F_1 + D_{1,b}))$

$F_6 = ((3D_{1,b}F_1F_2 + 3F_1^2F_2)F_3 - (-2D_{1,b}^3 - 6D_{1,b}^2F_1 - 6D_{1,b}F_1^2 - 2F_1^3)(1 - F_1^2/(D_{1,b} + F_1)^2)^{3/2} - (3D_{1,b}^2(F_1 + D_{1,b} + R_w) + 6D_{1,b}F_1(F_1 + D_{1,b} + R_w) + 3F_1^2(F_1 + D_{1,b} + R_w))F_5)/(3D_{1,b}F_1^2 + F_1(6D_{1,b}^2 - 6D_{1,b}F_2))$

$F_7 = (4D_{1,b}^3 - 3\pi D_{1,b}^2F_2 + F_1(12D_{1,b}^2 - 6\pi D_{1,b}F_2) + 12D_{1,b}F_1^2 + (-6F_1^2F_2 - 6D_{1,b}F_1F_2)F_3 + (-4F_1^3 - 12D_{1,b}F_1^2 - 12D_{1,b}^2F_1 - 4D_{1,b}^3)(1 - F_1^2/(D_{1,b} + F_1)^2)^{3/2} + (6F_1^2F_2 + 12D_{1,b}F_1F_2 + 6D_{1,b}^2F_2)F_5)/(6D_{1,b}F_1^2 - 12D_{1,b}F_1F_2)4D_{1,b}^3 - 3\pi D_{1,b}^2F_2 + F_1(12D_{1,b}^2 - 6\pi D_{1,b}F_2) + 12D_{1,b}F_1^2 + (-6F_1^2F_2 - 6D_{1,b}F_1F_2)F_3 + (-4F_1^3 - 12D_{1,b}F_1^2 - 12D_{1,b}^2F_1 - 4D_{1,b}^3)(1 - F_1^2/(D_{1,b} + F_1)^2)^{3/2} + (6F_1^2F_2 + 12D_{1,b}F_1F_2 + 6D_{1,b}^2F_2)F_5/6$

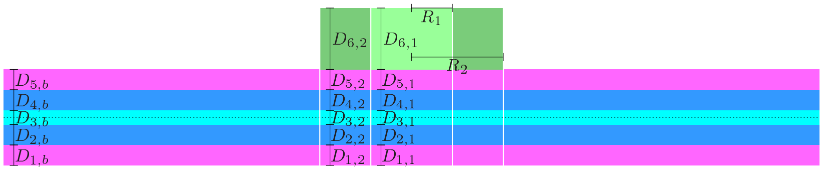

Figure S7: Anchored protein.

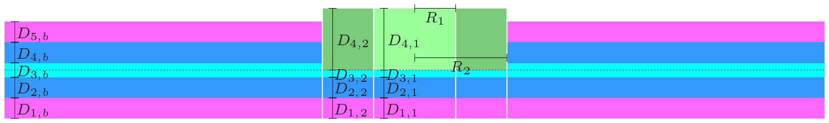

Figure S8: Monotopic protein.

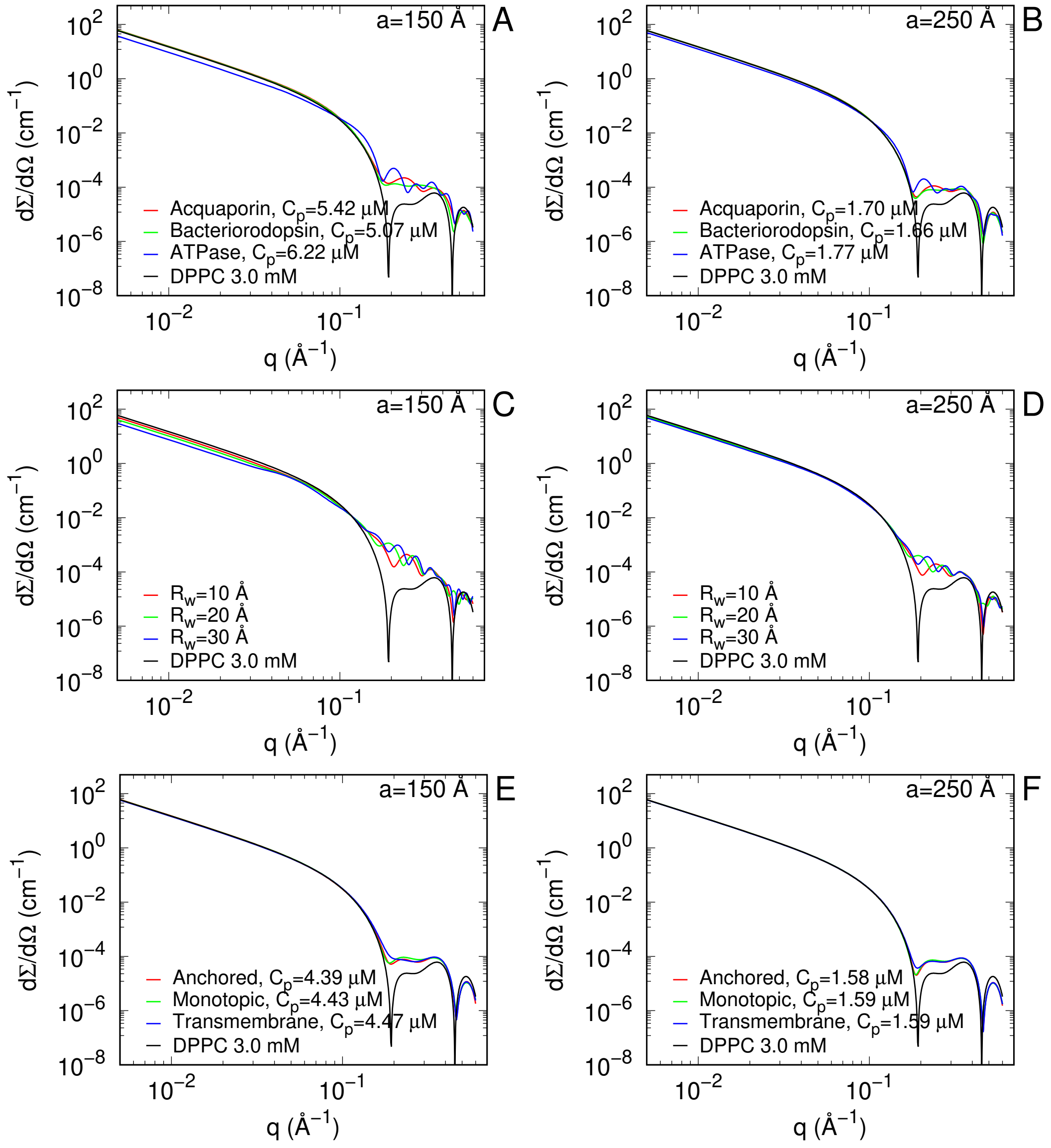

Figure S9: Simulated SANS curves at $x_D = 1$ of islands in a DPPC bilayer at $C_{\mathrm{DPPC}} = 3$ mM according to the present model. Panels A and B report three different transmembrane proteins (as indicated) with lattice parameter $a = 150$ Å (panel A) and $a = 250$ Å (panel B). Panels C and D report three different water pores (with indicated pore radius R_w) with lattice parameter $a = 150$ Å (panel C) and $a = 250$ Å (panel D). Panels E and F report three different positions of cytochrome-c (as indicated) with lattice parameter $a = 150$ Å (panel E) and $a = 250$ Å (panel F). In all cases the distortions parameter is $g_a = 0.3$

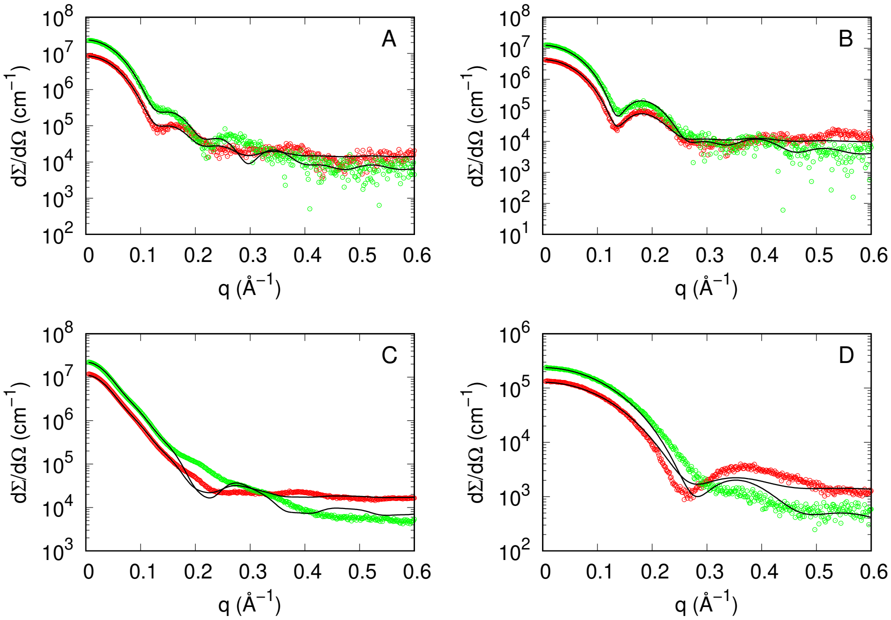

Figure S10: SAXS (red points) and SANS (green points) curves of aquaporin (panel A), bacteriorodopsin (panel B), ATPase (panel C) and cytochrome-c (panel D). Data have been simulated by using the SASMOL method from the pdb code 3D9S, 1FBB, 3WGV and 1GIW, respectively, and moved within an error bar proportional to $[d\Sigma/d\Omega(q)]^{1/2}$. Solid lines represent the simultaneous best fit obtained with the core-shell cylinder model (Eqs. S11-S14).

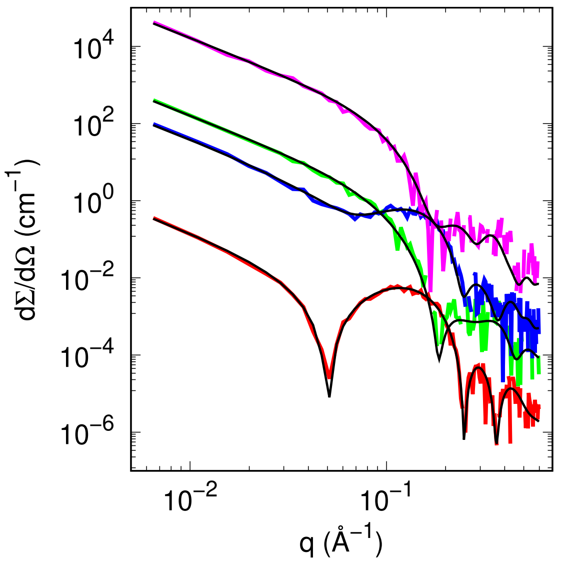

Figure S11: Best global-fit (black lines) of SAXS (red and blue lines) and SANS (green and magenta lines) curves of DPPC and aquaporin in DPPC according to the simulations shown in Fig. 5A and Fig. S9A of the SI. The simulated curves have been randomly moved by sampling from a Gaussian distribution with standard deviation proportional to $[d\Sigma/d\Omega(q)]^{1/2}$. Curves are vertically displaced for clarity.

Table S1: Best fit parameters of curves shown in Fig. S11 representing the transmembrane aquaporin in a DPPC bilayer. The length unit is Å. For X-ray, electron densities are expressed in e Å^{-3}. For neutrons, SLDs are expressed in 10^{-6} Å^{-2}. [(a)] input values are derived parameters.

		In	Out
R_1		5.78	15±1
δ		28.6	20.0±0.5
ϕ_e		0.904	0.87±0.04
ϕ_i		0.211	0.2±0.1
L	a	41.4	48±2
$D_{1,b}$		12.0	11.8±0.2
$D_{2,b}$		10.6	10.4±0.1
$D_{3,b}$		3.64	3.9±0.1
$\rho_{1,b,X}$		0.411	0.414±0.002
$\rho_{1,b,N}$		4.29	4.24±0.09
$\rho_{2,b,X}$		0.316	0.315±0.001
$\rho_{2,b,N}$		−0.618	−0.8189±0.0008
$\rho_{3,b,X}$		0.247	0.253±0.001
$\rho_{3,b,N}$		−0.126	−0.3±0.1
$\sigma_{1,b}$		2.56	3.0±0.2
$\sigma_{2,b}$		2.22	2.6±0.1
$\sigma_{3,b}$		1.74	1.6±0.2
g_a		0.300	0.39±0.03

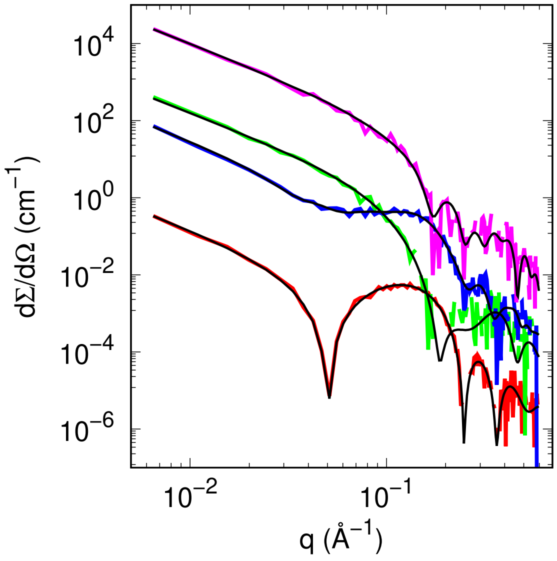

Figure S12: Best global-fit (black lines) of SAXS (red and blue lines) and SANS (green and magenta lines) curves of DPPC and ATPase in DPPC according to the simulations shown in Fig. 5A and Fig. S9A of the SI. The simulated curves have been randomly moved by sampling from a Gaussian distribution with standard deviation proportional to $[d\Sigma/d\Omega(q)]^{1/2}$. Curves are vertically displaced for clarity.

Table S2: Best fit parameters of curves shown in Fig. S12 representing the transmembrane ATPase in DPPC bilayer. The length unit is Å. For X-ray, electron densities are expressed in e Å^{-3}. For neutrons, SLDs are expressed in 10^{-6} Å^{-2}. $^{(a)}$ input values are derived parameters.

		In	Out
R_1		18.2	22±2
t		24.6	21±2
ϕ_e		0.0557	0.03±0.01
ϕ_i		1.00	0.77±0.09
L	a	107	118±8
$D_{1,b}$		12.0	12.6±0.2
$D_{2,b}$		10.6	10.4±0.2
$D_{3,b}$		3.64	3.5±0.3
$\rho_{1,b,X}$		0.411	0.409±0.002
$\rho_{1,b,N}$		4.29	4.12±0.09
$\rho_{2,b,X}$		0.316	0.314±0.002
$\rho_{2,b,N}$		−0.618	−0.7±0.2
$\rho_{3,b,X}$		0.247	0.247±0.004
$\rho_{3,b,N}$		−0.126	−0.2±0.3
$\sigma_{1,b}$		2.56	2.3±0.2
$\sigma_{2,b}$		2.22	2.1±0.4
$\sigma_{3,b}$		1.74	1.8±0.3
g_a		0.300	0.32±0.03

References

[1] Jacrot, B. The study of biological structures by neutron scattering from solution. *Reg. Prog. Phys.* **1976**, *39*, 911.
